# ^18^F-Labeled, PSMA-Targeted Radiotracers: Leveraging the Advantages of Radiofluorination for Prostate Cancer Molecular Imaging

**DOI:** 10.7150/thno.37894

**Published:** 2020-01-01

**Authors:** Rudolf A. Werner, Thorsten Derlin, Constantin Lapa, Sara Sheikbahaei, Takahiro Higuchi, Frederik L. Giesel, Spencer Behr, Alexander Drzezga, Hiroyuki Kimura, Andreas K. Buck, Frank M. Bengel, Martin G. Pomper, Michael A. Gorin, Steven P. Rowe

**Affiliations:** 1 Department of Nuclear Medicine, Hannover Medical School, Hannover, Germany.; 2 The Russell H. Morgan Department of Radiology and Radiological Science, Johns Hopkins University School of Medicine, Baltimore, MD, USA.; 3 Department of Nuclear Medicine, University Hospital Würzburg, Germany.; 4 Okayama University Graduate School of Medicine, Dentistry and Pharmaceutical Sciences, Okayama, Japan.; 5 Department of Nuclear Medicine, University Hospital Heidelberg, INF 400, 69120 Heidelberg, Germany.; 6 Department of Radiology and Biomedical Imaging, University of California, San Francisco, CA.; 7 Department of Nuclear Medicine, University Hospital Cologne, Germany.; 8 Department of Analytical and Bioinorganic Chemistry, Kyoto Pharmaceutical University, Kyoto, Japan.; 9 The James Buchanan Brady Urological Institute and Department of Urology, Johns Hopkins University School of Medicine, Baltimore, MD, USA.

**Keywords:** Radiofluorine, prostate-specific membrane antigen, prostate cancer, 18F, PSMA, PET, 68Ga, theranostics, radioligand therapy

## Abstract

Prostate-specific membrane antigen (PSMA)-targeted PET imaging for prostate cancer with ^68^Ga-labeled compounds has rapidly become adopted as part of routine clinical care in many parts of the world. However, recent years have witnessed the start of a shift from ^68^Ga- to ^18^F-labeled PSMA-targeted compounds. The latter imaging agents have several key advantages, which may lay the groundwork for an even more widespread adoption into the clinic. First, facilitated delivery from distant suppliers expands the availability of PET radiopharmaceuticals in smaller hospitals operating a PET center but lacking the patient volume to justify an onsite ^68^Ge/^68^Ga generator. Thus, such an approach meets the increasing demand for PSMA-targeted PET imaging in areas with lower population density and may even lead to cost-savings compared to in-house production. Moreover, ^18^F-labeled radiotracers have a higher positron yield and lower positron energy, which in turn decreases image noise, improves contrast resolution, and maximizes the likelihood of detecting subtle lesions. In addition, the longer half-life of 110 min allows for improved delayed imaging protocols and flexibility in study design, which may further increase diagnostic accuracy. Moreover, such compounds can be distributed to sites which are not allowed to produce radiotracers on-site due to regulatory issues or to centers without access to a cyclotron. In light of these advantageous characteristics, ^18^F-labeled PSMA-targeted PET radiotracers may play an important role in both optimizing this transformative imaging modality and making it widely available. We have aimed to provide a concise overview of emerging ^18^F-labeled PSMA-targeted radiotracers undergoing active clinical development. Given the wide array of available radiotracers, comparative studies are needed to firmly establish the role of the available ^18^F-labeled compounds in the field of molecular PCa imaging, preferably in different clinical scenarios.

## Introduction

As one of the most common malignancies in men in the United States and Europe, the incidence of metastatic prostate cancer (PCa) continues to rise 1-3. The ability to ascertain the presence and extent of metastatic disease has been significantly increased through the introduction of imaging agents targeting prostate-specific membrane antigen (PSMA) which provide superior diagnostic performance compared to alternative techniques, with a calculated positive predictive value of >95% 4-6. These PSMA-targeted PET radioligands have not only been found to have diagnostic accuracy for visualizing sites of PCa, but have also paved the way for a theranostic approach utilizing alpha/beta-labelled agents, in a manner similar to peptide receptor radionuclide therapy for neuroendocrine tumors 7-10. In this regard, the theranostic principle is based on the concept of a predictive biomarker (e.g. sufficient uptake on a PSMA-targeted PET scan in putative sites of disease), followed by an indivualized treatment with a therapeutic agent, e.g. by targeted ß^-^ treatment using ^177^Lu-labeled PSMA inhibiting compounds 11, 12.

In general, a PET-based radionuclide for imaging PCa should inherit several key properties to generate good imaging quality and to satisfy the needs of the treating urologist: the compound should (I.) have a high affinity towards a target on PCa cells providing very high tumor-to-background ratios, (II.) identify even very small volume sites of disease, (III.) have a biodistribution favorable to the detection of typical sites of disease such as the prostate bed, pelvic lymph nodes, and bone, (IV.) work reliably among a large variety of different clinical contexts including initial staging, restaging at the time of biochemical recurrence, radiation or surgery planning, (V.) have a high radiochemical yield enabling for a high throughput of patients, (VI.) have been extensively validated in a (pre-)clinical setting, (VII.) outperform current state-of-the-art imaging approaches, and (VIII.) allow for endoradiotherapy by labelling structurally related agents with ^177^Lu, ^90^Y, or alpha-emitting radionuclides.

To date, ^68^Ga-labeled imaging agents are by far the most commonly used PSMA-targeted PET radiotracers in clinical practice 13. However, recent years have witnessed the beginning of a shift from ^68^Ga- to ^18^F-labeled compounds 14. The latter radionuclide offers several advantages compared to ^68^Ga including: a.) flexibility in study design with potential for delayed imaging protocols due to a longer half-life, b.) lower positron energy with resultant short positron range in tissue and improved contrast and noise, and c.) potential for distribution by commercial vendors through existing shipping networks 15, 16. Notably, such dispatch systems from central cyclotron facilities to smaller hospitals have led to remarkable cost-savings for [^18^F]FDG 17. Thus, if a more widespread adoption of PSMA-targeted PET imaging is to be pursued in the near future, ^18^F-labeled radiotracers may play a pivotal role in meeting this increasing demand in patient care. In addition to that, recent studies have already suggested that the theoretical improved imaging possible with ^18^F- compared to ^68^Ga-labeled PSMA agents may already have an impact on patient care, such as in subjects with biochemically-relapsed PCa 18-20. Nonetheless, ^18^F labeling chemistry has also several drawbacks, e.g. time-consuming labeling procedures (e.g. indirect labeling methods instead of direct fluorination methods using a single precursor) 21, challenging radiosynthesis (e.g. varying chemoselectivity for the incorporation of ^18^F into compounds such as peptides) 22, installation and maintenance of a costly cyclotron, and limited experience with theranostic approaches based on ^18^F-labeled compounds as the diagnostic agents 23.

**Figure 1:**
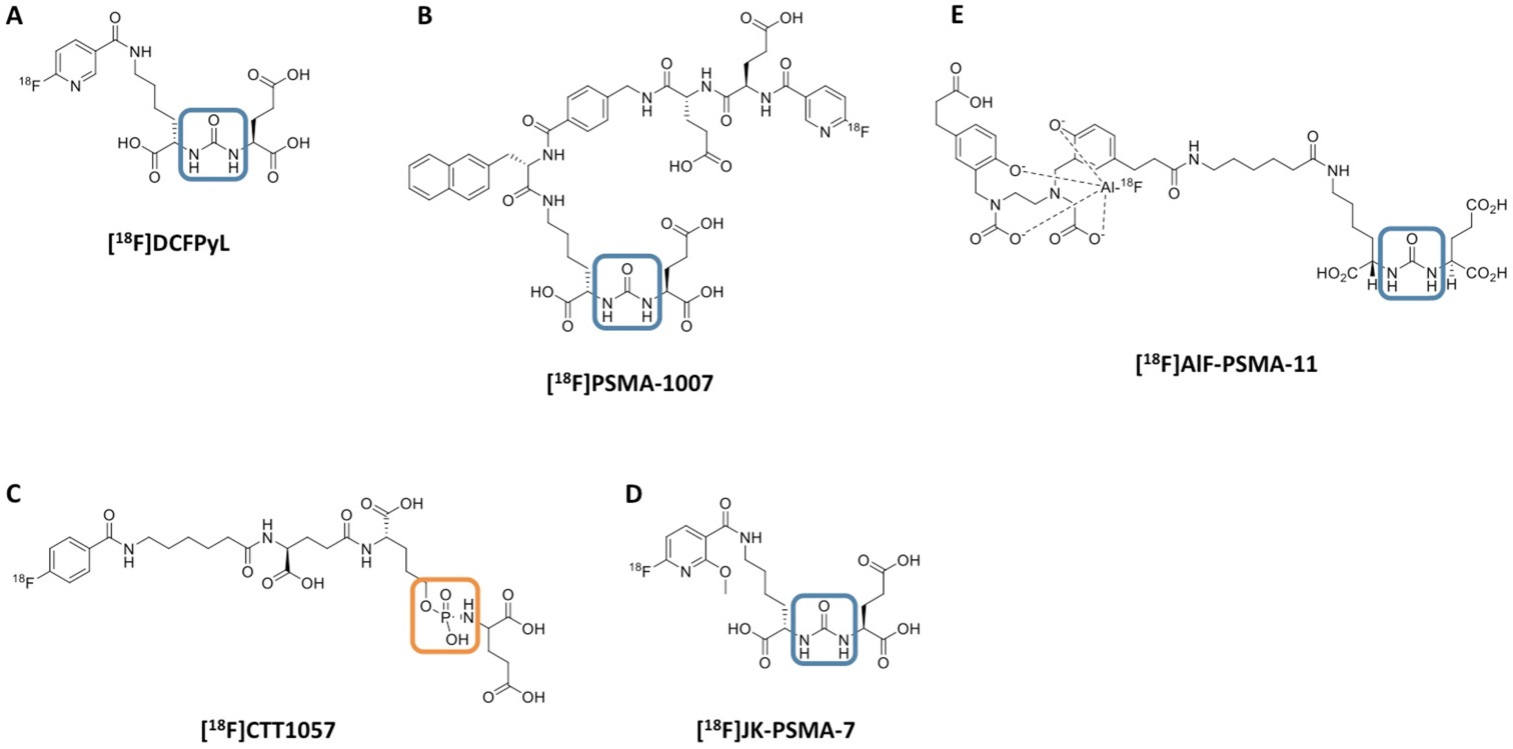
** Chemical structure of ^18^F-labeled radiotracers.** [^18^F]DCFPyL (A), [^18^F]PSMA-1007 (B), [^18^F]CTT1057 (C), (D) [^18^F]JK-PSMA-7 and (E) [^18^F]AIF-PSMA-11. The urea backbone of (A), (B), (D) and (E) is marked in blue, while the phosphoramidate of [^18^F]CTT1057 in (C) is highlighted in orange. Modified from *Behr* et al. 32, © by the Society of Nuclear Medicine and Molecular Imaging, Inc.

However, given the increasing use of ^18^F radiochemistry for molecular imaging in PCa, we aimed to provide an overview of recently introduced ^18^F-labeled radiotracers for PSMA-targeted PET imaging. Older, first-generation, ^18^F-labeled radiotracers for PSMA including [^18^F]DCFBC and the phosphonomethyl compound BAY 1075553 will not be the focus of this review of more-recently-developed agents, but we mention them here to acknowledge the important role of such compounds in providing early evidence of the feasibility of imaging PSMA with ^18^F-labeled agents 24-30. This review will focus on newer radiotracers including clinical development of a number of agents, such as the clinically established radiotracers [^18^F]DCFPyL and [^18^F]PSMA-1007 (Part I) and the recently introduced agents [^18^F]CTT1057, [^18^F]-FSU-880, [^18^F]JK-PSMA-7 and [^18^F]AlF-PSMA-11 (Part II) 14, 31-35. Figure 1 illustrates the chemical structures of selected ^18^F-labeled compounds 32.

### ^18^F vs. ^68^Ga radiochemistry in molecular imaging of PCa

Compared to ^18^F, ^68^Ga has multiple drawbacks:

1.) The lower positron yield (^68^Ga, 89.14% vs. ^18^F, 96.86%) and higher positron energy (^68^Ga, 1,899 keV vs. ^18^F, 633 keV) of ^68^Ga may impact image quality and degrade diagnostic yield 36. Among other physical properties, those factors contribute to the partial volume effect, which in turn has a major impact on semi-quantification, e.g. by intra- or inter-lesion comparisons using standardized uptake values (SUV) 37. For instance, the image spatial resolution is inferior with ^68^Ga compared to ^18^F (2.4 vs 1.4 mm in all directions) 38. Given the presence of multiple single-photon emissions, imaging noise is also increased with ^68^Ga 37.

2.) In light of the large variety of commercially-available ^68^Ga generators, varying properties of these generators (e.g. hydrochloric acid concentration, eluate processing procedures, or cationic/anionic exchange cartridges) have to be taken into account 39. The ability to mass produce ^18^F-labeled PSMA inhibitors will provide a supply of PET agent that would saturate any demand for such agents. Similar supply cannot be met with a ^68^Ga generator-dependent PET agent 36.

3.) Delivery of ^18^F-labeled radiopharmaceuticals from distant suppliers expands the availability of PET radiopharmaceuticals in smaller hospitals operating a PET center remote from a cyclotron facility 17. This is particularly important given the growing demand for PSMA-targeted PET scans around the globe. However, for ^68^Ga generators, a relatively high throughput of patients is needed to reduce the cost per injected dose 40. Thus, commercial sources for ^18^F-labeled compounds can offer a cost-effective alternative to in-house cyclotrons, as has been proven for [^18^F]FDG 41. In the U.S., Medicare spending is anticipated to double between 2010 and 2030 to approximately $1.2 trillion and thus, escalating health care expenses increase the demand for such effective cost containment strategies 42. Purchasing [^18^F]FDG from a production supplier located 1200 miles away, *Durcharme* et al. have not only reported on the feasibility of incorporating such a concept in the clinic, but also summarized practical aspects when receiving ^18^F-labeled agents from a distant supplier 17. In addition, compared to ^18^F, the shorter half-life of ^68^Ga will limit the shipping range of ^68^Ga-labeled PSMA-targeted radiotracers to remote PET facilities.

4.) The longer half-life of ^18^F allows for delayed imaging protocols. Using [^68^Ga]PSMA-HBED-CC, *Hohberg* and co-workers reported on a higher lesion detection rate for a later imaging time-point as compared to early imaging (1h vs 3h post-injection), in particular for patients with low PSA levels 43. *Schmuck* et al. have also shown that in patients with biochemical recurrence or PSA persistence after primary therapy for PCa, tumor-to-background ratios have increased at later imaging time-points using [^68^Ga]PSMA I&T 44. ^18^F has a longer half-life and thus, one may hypothesize whether even more delayed imaging protocols with ^18^F-labeled PSMA radiotracers may also further increase overall detection rate. This also applies to increased acquisition times, which improves overall imaging quality by reducing image noise in PCa molecular imaging 45.

5.) The success of ^18^F-labeled, PSMA-targeted PET agents is most likely owed to the widespread adoption of the most commonly used radiotracer in oncology, namely [^18^F]FDG 46. Thus, for ^18^F-labeled imaging agents for PCa, existing cylotrons, infrastructure, and protocols for production, handling, and transport of ^18^F can be effectively utilized.

Nonetheless, several drawbacks of ^18^F-labeled compounds have to be considered, in particular when compared to current ^68^Ga-PSMA PET compounds:

1.) The time-consuming and challenging ^18^F radiochemistry is an important potential limitation 23, although centralized production of ^18^F-labeled compounds, as opposed to onsite production, would substantially mitigate this drawback.

2.) Given the increasing availability of ^68^Ge^/68^Ga generators with a half-life of 271 days, an on-site cyclotron is not necessary for having access to ^68^Ga for labeling radiopharmaceutical precursors 37.

3.) ^68^Ga-labeled PSMA specific compounds are better evaluated based upon their long-standing use in daily patient care. For instance, *Fendler* et al. recently evaluated [^68^Ga]PSMA-11 in a prospective single-arm trial enrolling 635 men 47.

4.) To date, theranostic experiences with ^177^Lu-labeled PSMA inhibitors are based on sufficient uptake in a preceding ^68^Ga-PSMA PET scan 12.

5.) The costs for 70 MeV cyclotron can be >$13,000,000, while commercial automated ^68^Ga-labeling snythesis units cost approximately $10,000 - $50,000 48, 49 (Table 1).

## Part I: Clinically established ^18^F-labeled radiotracers

### [^18^F]DCFPyL

First, *Szabo* and co-workers reported on nine patients with known metastatic PCa. Apart from an acceptable safety profile, increased radiotracer accumulation was appreciated in presumed primary and metastatic sites of disease, along with an effective radiation dose comparable to [^18^F]FDG (0.0319 mGy/MBq after injection of 370 MBq). In descending frequency, the highest radiation dose was recorded in the kidneys (most likely caused by specific binding), followed by the urinary bladder wall, the submandibular glands and the liver. Compared to other ^18^F-labeled PSMA agents, the latter finding of less hepatic uptake may play a role for an increased detection rate of liver lesions attributable to PCa 14 and may aid in the identification of high retroperitoneal lymph nodes.

**Figure 2:**
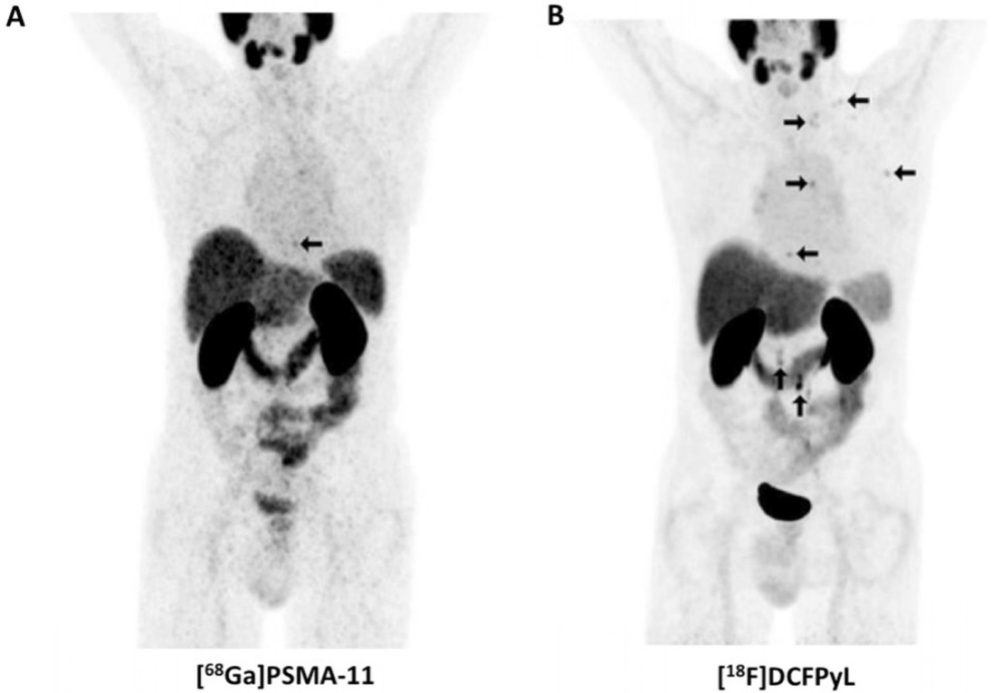
Head-to-head comparison of maximum intensity projections of [^68^Ga]PSMA-11 and [^18^F]DCFPyL in a patient with rising levels of prostate-specific membrane antigen. For [^18^F]DCFPyL, additional PSMA-positive supradiapragmatic lesions are noted. Modified from *Dietlein* et al. 18, © the authors (2015), published under the terms of the Creative Commons Attribution 4.0 International supradiaphragmatic (http://creativecommons.org/licenses/by/4.0/).

Not surprisingly, [^18^F]DCFPyL outperformed other molecular imaging agents (^99m^Tc-methylene diphosphonate bone scan, Na^18^F PET/CT) for lesion detection in patients with PCa. In a 45-year old man with suspected oligometastatic PCa, 87 definitive sites of non-physiologic [^18^F]DCFPyL accumulation were detected, while planar bone scan detected only 12 supicious sites and Na^18^F PET/CT 39 lesions 50. The same research group also reported on the superiority of [^18^F]DCFPyL compared to conventional imaging on a larger basis. In a secondary analysis of the first nine patients imaged with [^18^F]DCFPyL, 138 definitive sites of [^18^F]DCFPyL uptake were recorded, while only 1 was classified as equivocal. This was in contradistinction to CT-and-bone-scan based disease patterns (30 definitive, 15 equivocal). Taking into account intra-patient clustering effects, lesions which have been rated as negative/equivocal on conventional imaging would be positive on [^18^F]DCFPyL in the vast majority of the cases (0.72, based on a generalized estimating equation regression analysis) 5. In addition, [^18^F]DCFPyL has also been tested in a head-to-head comparison with its ^68^Ga-labeled counterpart, namely [^68^Ga]PSMA-11. In 14 patients with biochemically recurrent disease, the ^18^F-labeled radiotracer identified more sites of disease in three patients and those findings were also further corroborated semiquantitatively (higher maximum SUV (SUV_max_) and tumor-to background ratios). The results of this study suggest [^18^F]DCFPyL is an attractive alternative to ^68^Ga-labeled compounds (Figure 2) 18.

Given the promising data comparing [^18^F]DCFPyL to a ^68^Ga-based imaging agent, *Giesel* et al. performed a pilot study investigating the novel ^18^F-labeled PSMA-targeted PET compound [^18^F]PSMA-1007 with [^18^F]DCFPyL. In the twelve selected, treatment-naïve patients, no significant differences were recorded regarding visual or semiquantitative assessment (SUV_max_) of putative sites of disease. However, distinct characteristics were noted between both radiotracers. In an organ-to-background analysis, [^18^F]DCFPyL had significant higher radiotracer accumulation in the urinary system and lacrimal glands, which may hamper diagnostic accuracy in small lymph nodes in the (lower) pelvis. This was in contradistinction to [^18^F]PSMA-1007, which demonstrated increased uptake levels in the muscle, submandibular and sublingual glands, spleen, pancreas, gallbladder, and liver. The latter organ uptake may decrease the level of certainty of a reader that an equivocal finding in the liver is a site of disease, in particular without a cross-sectional imaging correlate 51.

*Wondergem* et al. compared a 60 vs. 120 min time-to-scan interval using [^18^F]DCFPyL and showed that >38% of all investigated patients had more suspicious lesions when the later imaging protocol was applied. Notably, this led to a change in TNM staging in >9% 52. If there is widespread adoption of ^18^F-labeled, PSMA-targeted radiotracers for PCa imaging or even an incorporation of those agents in clinical guidelines, such observations are of utmost importance for establishing adequate imaging protocols. *Rousseau* and co-workers tested [^18^F]DCFPyL in a prospective setting in 130 subjects and reported on detection of recurrent PCa in 78% for PSA levels of ≥0.5-<1 ng/ml and 92% for ≥2.0 ng/ml 53. Thus, compared to a recent prospective trial using [^68^Ga]PSMA-11, detection rates with [^18^F]DCFPyL were substantially higher, in particular in patients with low-level PSA ([^68^Ga]PSMA-11: 0.5 - <1 ng/ml, 57% and 2.0 ng/ml, 86%) 47, 53. These findings further suggest that the inherent advantages of ^18^F-labeled radiotracers over ^68^Ga-labeled agents may translate into clinical practice. Notably, management plans changed in >87% after conducting an [^18^F]DCFPyL scan with a change in treatment intent in >65% 53.

Many pitfalls for both ^68^Ga- and ^18^F-labeled PSMA-targeting radiotracers have been reported since their routine introduction in the clinic 54. These include, but are not limited to: benign sites of radiotracer accumulation (e.g. ganglia which can be misinterpreted as lymph node metastases or healing fractures as bone lesions), benign vascular tumors (hepatic hemangioma), pulmonary involvement (sarcoidosis or tuberculosis) or even non-prostatic malignant tumors such as renal cell carcinoma 55. To address these manifold pitfalls, framework systems for PSMA-targeted PET imaging have been recently introduced, and such reporting systems may meet the need to harmonize interpretation of PSMA PET-based findings 56, 57. Readers with different levels of experience each read 50 [^18^F]DCFPyL PET/CTs and utilized the PSMA reporting and data system (PSMA-RADS) framework to classify up to five target lesions. Notably, using such a structured reporting system, the majority of the PET studies were assigned to the highest PSMA-RADS score (PSMA-RADS-4 or -5), which indicates that the readers were convinced that PCa was either highly likely or certainly present. Thus, this study further suggests the high sensitivity and specificity of [^18^F]DCFPyL for assessing sites of disease solely on a visual level. Moreover, the high concordance rate, even among readers with different levels of experience, suggests that this radiotracer is nearing readiness to be implemented in the collection of data for larger prospective trials 58.

Beyond its potentially pivotal role in PCa imaging, an extensive body of literature has also reported on the utility of this radiotracer for non-PCa malignancies and malignant-like conditions, e.g. for renal cell carcinoma, oncocytoma, and high-grade gliomas 59-63. These findings render this radiopharmaceutical an attractive compound for other tumor entities, not only for diagnostic purposes, but also for selecting patients for radioligand treatment (RLT). However, prior to an implementation as a potential therapeutic radiotracer, reproducible methods for a reliable (peri)therapeutic dosimetry are indispensable, which in turn allows for maximizing therapeutic efficacy but minimizing potential harm 64. Thus, *Plyku* et al. calculated the absorbed doses of [^18^F]DCFPyL to the lacrimal and salivary glands based on Monte Carlo models, which are also known as the potentially dose-limiting organs for RLT and thus, this study may lay the groundwork for an application of compounds structurally related to [^18^F]DCFPyL in a treatment setting 65.

The OSPREY trial (“A PrOspective Phase 2/3 Multi-Center Study of [^18^F]DCFPyL PET/CT Imaging in Patients With PRostate Cancer: Examination of Diagnostic AccuracY”, ClinicalTrials.gov Identifier: NCT02981368) investigated 385 patients and can provide additional insights into the diagnostic performance of ^18^F-DCFPyL. In this study, patients with either clinically localized PCa and patients with metastatic PCa were included in a prospective trial. The primary endpoint is the accuracy of [^18^F]DCFPyL to either detect PCa in pelvic lymph nodes in patients scheduled to undergo radical prostatectomy or the detection rate of metastatic PCa in patients willing to undergo biopsy 66. OSPREY is being followed by another trial known as CONDOR (“A Phase 3, Multi-Center, Open-Label Study to Assess the Diagnostic Performance and Clinical Impact of ^18^F-DCFPyL PET/CT Imaging Results in Men With Suspected Recurrence of Prostate Cancer”, ClinicalTrials.gov Identifier: NCT03739684) that is examining the role of [^18^F]DCFPyL in biochemical recurrence in men with negative or equivocal conventional imaging.

### [^18^F]PSMA-1007

**Figure 3:**
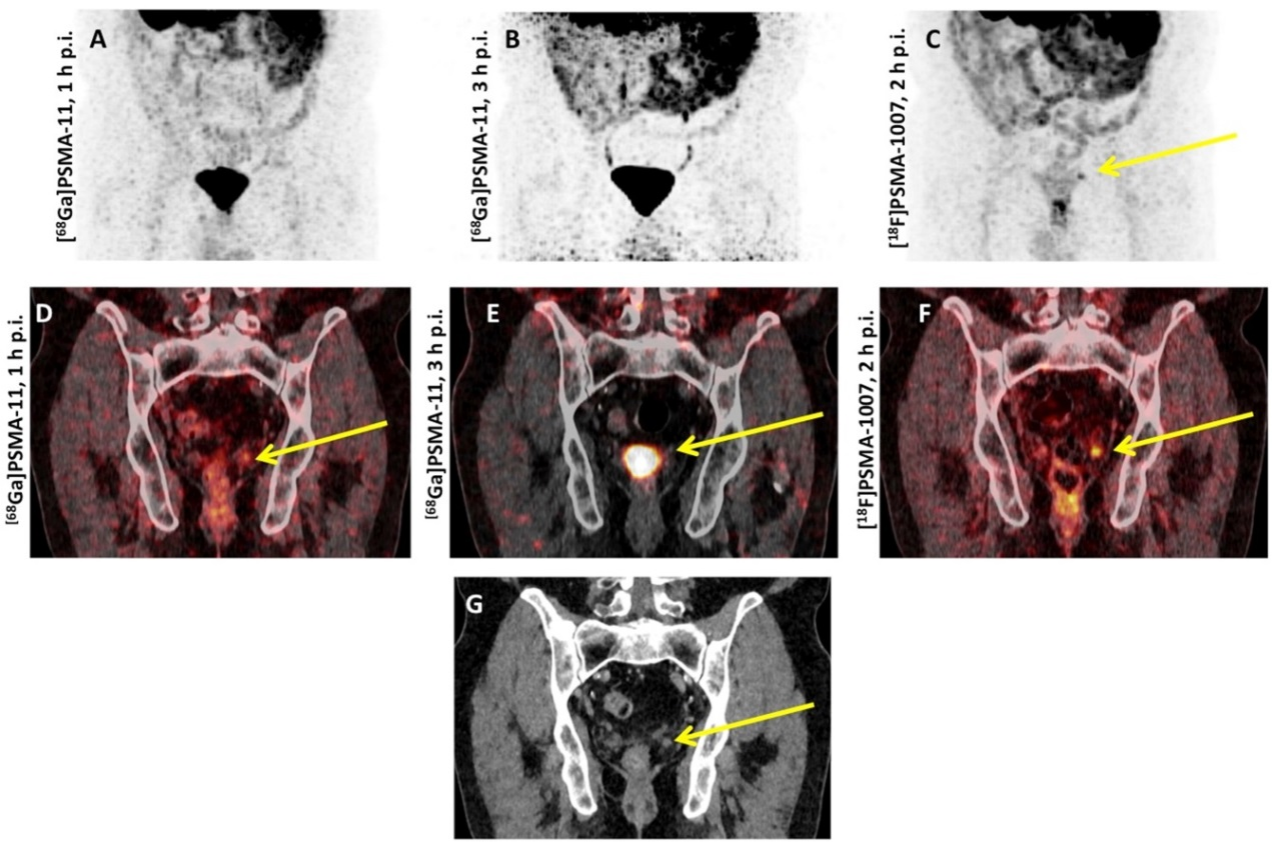
** Head-to-head comparison of [^68^Ga]PSMA-11 vs. [^18^F]PSMA-1007 PET/CT.** Biochemical relapse after prostatectomy, localized in the fossa of the seminal vesicle on the left. (A,D) early and (B,E) late [^68^Ga]PSMA-11 PET/CT scans which showed an equivocal finding. The residual activity of [^68^Ga]PSMA-11 3 hours p.i. was too low for a final interpretation (E). The additionally performed [^18^F]PSMA-1007 revealed the relapse with a PSMA overexpression, demonstrated on the maximal intensity projection (C) and on the coronal tomogram (F). The relapse showed a correlate on the low-dose CT (G).

Spearheaded by *Giesel* et al., [^18^F]PSMA-1007 was first tested in a patient with biochemically recurrent PCa and this imaging agent detected micrometastases along the retroperitoneum and iliac arteries 67. Thereafter, this compound was further evaluated in a Phase 1 trial using three healthy volunteers and ten PCa patients (injection-to-imaging intervals, 1 h and 3 h). First and foremost, the authors reported on no adverse events and the mean effective dose was comparable to other ^18^F-labeled PSMA PET agents (injected amount of radiotracer activity, 200-250 MBq). The notably reduced renal excretion of [^18^F]PSMA-1007 may further increase diagnostic accuracy for small lymph node metastases in the pelvis (particularly if located along the ureters) or to assess lesions below/adjacent to the urinary bladder, i.e. local recurrence (clearance via urinary tract for [^18^F]PSMA-1007 during the first 2 h p.i., 1.2% of the injected activity vs. 11% for ^18^F-DCFPyL) 14, 31. Beyond a report on visual assessment, the authors also performed a histopathological comparison of patients who underwent prostatectomy and the diagnostic findings were validated by examinations of harvested tumor tissue 31. Based on these encouraging findings, *Rahbar* et al. evaluated 100 patients with biochemical relapse in a retrospective setting and distinctive detection frequencies among patients with different PSA levels were noted, with rates of positive scans as follows: 86%, 89%, 100% and 100% among patients with PSA levels of ≤0.5, 0.51-1.0, 1.1-2.0 and > 2.0 ng/ml, respectively 68. *Giesel* et al. provided further insights on the diagnostic efficacy of [^18^F]PSMA-1007 in >250 patients suffering from biochemical recurrence after radical prostatectomy: Further improvements in the overall detection rate were recorded, in particular in low and ultra-low PSA levels of 0.5-<1 and 0.2-<0.5 ng/mL with detection rates of 74.5% and 61.5%, respectively 69. *Rahbar* and coworkers also reported on the superior diagnostic performance of [^18^F]PSMA-1007 in comparison with [^68^Ga]PSMA-11, in particular for segregating local recurrence from physiological radiotracer excretion 70. Similar to [^18^F]DCFPyL, [^18^F]PSMA-1007 has an increased lesion detection rate at 120 min p.i. compared to an early imaging protocol 60 min p.i., along with a significant increase in SUV_max_ 71 (Figure 3). The recently launched “[^18^F]PSMA-1007 Global Initiative“ aims to establish further phase I/II trials and these results may reveal insights into the added value of this imaging agent among high-risk individuals 72. Recently, *Rauscher* et al. reported on a matched-pair comparison in patients undergoing either [^68^Ga]PSMA-11 or [^18^F]PSMA-1007 PET/CT. For the latter agent, a higher number of lesions with quantitatively increased radiotracer accumulation attributed to benign lesions were found, which further emphasizes the need of increasing reader's confidence to identify such pitfalls 73. Thus, incorporation of structured reporting systems may be of relevance for an accurate scan interpretation, in particular when reading an ^18^F-labeled PSMA PET scan 57.

Table 2 provides a comparison of the performance of [^18^F]DCFPyL and [^18^F]PSMA-1007 to ^68^Ga-labeled PSMA-targeting radiotracers.

## Part II: Recently introduced ^18^F-labeled radiotracers

### [^18^F]CTT1057

*Radiochemistry.* In brief, for the production of [^18^F]CTT1057, succinimidyl-^18^F-fluorobenzoate is coupled to the primary amine precursor CTT1298. Production was carried out on ORA Neptis® Perform synthesizer (Optimized Radiochemical Applications, Philippeville, Belgium) 32.

*Preclinical Investigations.* While both [^18^F]DCFPyL and [^18^F]PSMA-1007 are based around an urea moiety that binds in the PSMA active side, the novel ^18^F-labeled compound CTT1057 is based on a phosphoramidate core (Figure 1). Incorporating such a phosphoramidate scaffold, the binding to PSMA is irreversible (based on IC50 determinations), which may result in improved target-to-background ratios. A structurally related compound was investigated in [PSMA+] xenografts and demonstrated good imaging qualities, along with high uptake in sites of disease and rapid non-target clearance 74. Notably, [^177^Lu]CTT1403 is based on the same binding scaffold like its PET counterpart CTT1057 and the therapeutic efficacy of the ^177^Lu-labeled compound has already been tested in a preclinical setting 75.

**Figure 4:**
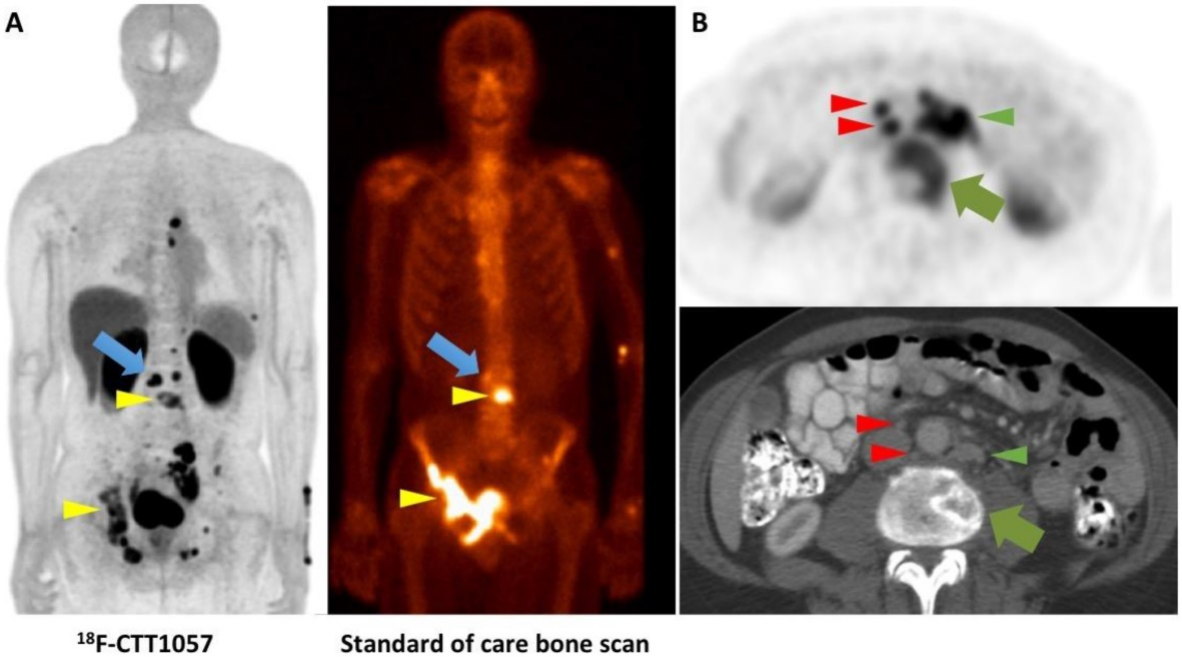
** Head-to-head comparison of [^18^F]CTT1057 PET study vs. standard of care bone scan vs. conventional CT.** (A) [^18^F]CTT1057 maximum intensity projection (MIP) PET (left) with several matching PSMA avid osseous lesions on standard of care bone scan (right, yellow arrowheads), which can be seen on both imaging modalities. However, a PSMA avid lesion on [^18^F]CTT1057 in the skeleton has no clear bone scan correlate (blue arrow). (B) Axial [^18^F]CTT1057 PET (upper row, red arrowheads) highlights a 3 mm lymph node that is not enlarged by size criteria on conventional CT (lower row), but has marked [^18^F]CTT1057 uptake. In addition, further enlarged PSMA avid retroperitoneal lymph nodes can be detected (red arrowheads), along with PSMA avid lytic osseous metastases (green arrows).

*Clinical Investigations.* Using a 3-tesla time-of-flight PET/MR system, *Behr* et al. recently reported on the first-in-human study of [^18^F]CTT1057. The authors indicated an acceptable safety profile and an average total effective dose of 0.023 mSv/MBq with the urinary bladder wall having the highest uptake. Notably, small bowel activity was rather minimal, which renders this agent a favorable diagnostic agent to assess sites of disease in the mid-abdomen, e.g. small lymph nodes. The effective dose from [^18^F]CTT1057 was similar to [^68^Ga]PSMA-11 and [^18^F]PSMA-1007, but slightly higher than [^18^F]DCFPyL 14, 31, 76. In 5 patients that were scheduled for imaging with [^18^F]CTT1057 followed by prostatectomy, 4 out of 5 patients had [^18^F]CTT1057 positive lesions corresponding to pathology-proven PCa, while one patient had no focal prostatic uptake. In a second cohort suffering from metastatic castration-resistant PCa, the overall detection rate was higher with the novel compound compared to conventional imaging modalities, including bone scan and CT (Figure 4). Again, a late imaging protocol (90 min p.i.) increased the overall detection rate. In this cohort, all patients had definitive local therapy and the lowest PSA level was 0.7 ng/ml. Thus, the detection rate may be comparable to clinically established agents, such [^18^F]PSMA-1007, and [^18^F]DCFPyL 32, 53, 68, but future trials will investigate the accuracy of this agent in sites of disease, in particular in patients with low PSA levels 32.

### [^18^F]FSU-880

*Radiochemistry.* After having synthesized a radioiodinated urea compound targeting PSMA ([^123^I]IGLCE), *Harada* et al. substituted the iodobenzamido group by a fluorobenzamido using [^18^F]SFB, which led to the development of [^18^F]FSU-880 77.

*Preclinical Investigations.* After synthesizing four ^18^F-labeled asymmetric urea compounds on the basis of [^18^F]SFB (including [^18^F]FSU-880), these radiotracers were investigated in a biodistribution study using human prostate cancer xenograft-bearing mice (with [^18^F]DCFPyL serving as reference standard). Only [^18^F]FSU-880 demonstrated similar biodistribution profiles to [^18^F]DCFPyL, along with rapid blood clearance, increased accumulation in [PSMA+] LNCaP tumors, low accumulation in the skeleton, almost exclusive excretion from the kidneys and moderate to low liver uptake 77.

**Figure 5:**
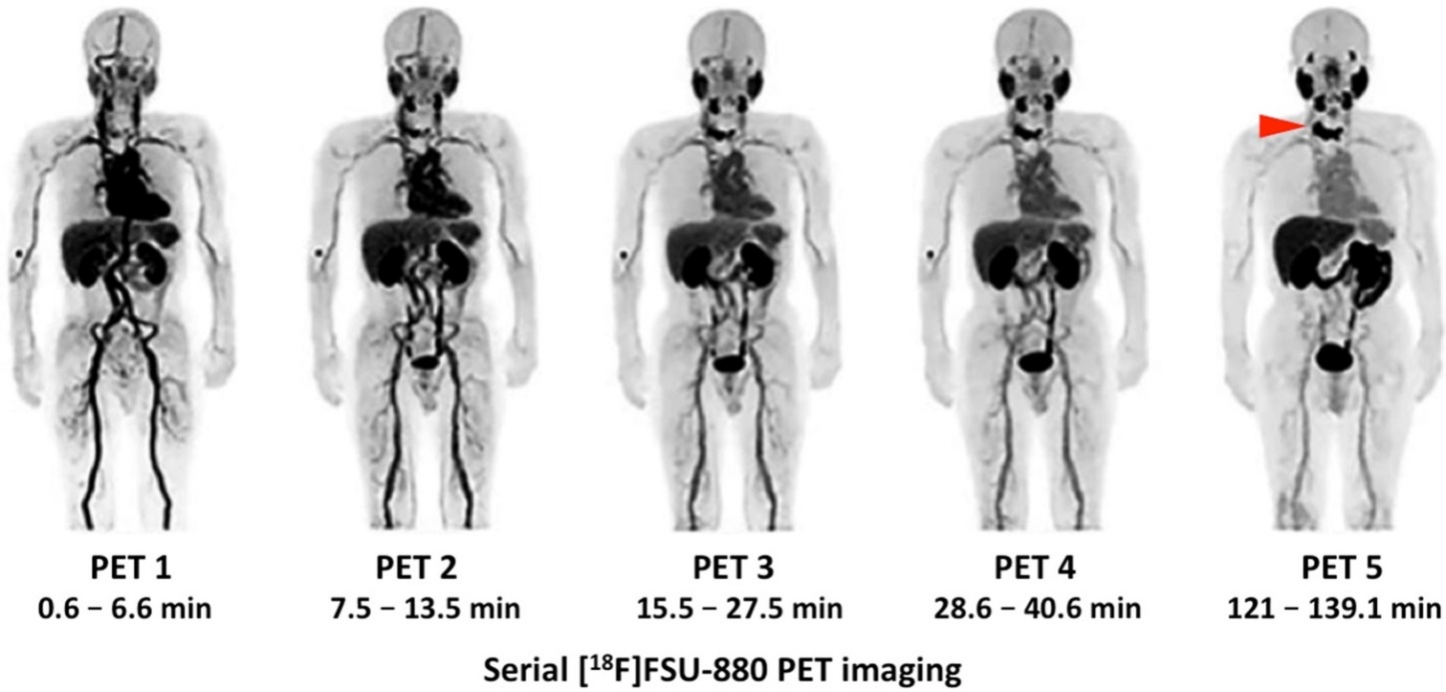
** Serial [^18^F]FSU-880 PET imaging.** Whole-body distribution of [^18^F]FSU-880 in a patient suffering from prostate cancer with known metastatic disease. Maximum intensity projections of 5 serially performed [^18^F]FSU-880 PET studies (up to 2 h after radiotracer injection) are displayed. Uptake can be noted in a bone metastasis of the upper thoracic vertebrae, which has been already evident in PET 1, but can be even more clearly seen 2 h after administration of [^18^F]FSU-880 (PET 5, red arrow). Such findings further emphasize the importance of late imaging time-points in ^18^F-labeled PSMA imaging. Modified from *Saga* et al. 33, © the authors (2018), published under the terms of the http://creativecommons.org/licenses/by-nc/4.0/ license.

*Clinical Investigations.* In light of these encouraging findings, [^18^F]FSU-880 was tested in six PCa patients with known metastatic disease. Radiotracer accumulation was noted in all sites of disease in the majority of the patients (5/6, with one patient under abiraterone acetate showing no uptake). No adverse events were recorded. The physiological biodistribution was similar to those of other small-molecule PSMA-targeting probes, with the kidneys receiving the highest dose, followed by the liver. Notably, four subsequent scans have been performed after injection of the radiotracer, with the final scan after 2 h. Again, a time-dependent increase in uptake was noted, with higher SUV_max_ and tumor-to-blood ratios in the primary and metastatic lesions at later imaging time-points 33. However, all of the included subjects had a Gleason score ≥7 and increased PSA-levels of 11.12 - 487 ng/ml. Thus, further studies are needed to investigate the performance of [^18^F]FSU-880 in patients with low PSA levels, preferably in a head-to-head comparison with the clinically established agents [^18^F]PSMA-1007 and [^18^F]DCFPyL 33. Figure 5 shows such serially performed PET studies with [^18^F]FSU-880 in a patient with bone involvement.

### [^18^F]JK-PSMA-7

*Radiochemistry.* In brief, [^18^F]JK-PSMA-7 was prepared using a two-step reaction: In the first step, the radiolabeled active ester was produced by the nucleophile reaction of ^18^F with 2-methoxy-*N*,*N*,*N*-trimethyl-5-((2,3,5,6-tetrafluoro-phenoxy) carbonyl) pyridine-2-aminium-trifluoromethanesulfonate (TFP-OMe-OFT) to generate the ester 2,3,5,6-tetrafluorophenyl-6-([^18^F]fluoro)-2-methoxy-nicotinate ([^18^F]FPy-OMe-TFP). In the second step, 4.6 ± 0.1 mg ((S)-5-amino-1-carboxypentyl)-carbamoyl)-L-glutamic-acid (LYS-GLU) was added to [^18^F]FPy-OMe-TFP and subsequently incubated at 45°C for 6 minutes. The final product, [^18^F]JK-PSMA-7, was purified by Solid-Phase Extraction (OASIS HLB) and formulated in saline. This reaction provided [^18^F]JK-PSMA-7 in high radiochemical yield, up to 40%, and in a high radiochemical purity (> 95%); the specific activity was 75 - 120 GBq/μmol. The detailed procedure for the radiosynthesis using the “minimalist” protocol is described elsewhere 34.

*Preclinical Investigations.* As alluded to earlier, [^18^F]JK-PSMA-7 has been compared to [^18^F]DCFPyL, [^18^F]PSMA-1007 and [^68^Ga]PSMA-11 in a preclinical setting. *Zlatopolskiy* et al. prepared eight ^18^F-labeled PSMA agents and the most promising one ([^18^F]JK-PSMA-7) was evaluated in peripheral ganglia of rats. As an underlying rationale, human ganglia are known to accumulate PSMA-targeted imaging agents and this model mimics small PSMA-expressing lesions comparable to the size of small lymph nodes (diameter of up to 3.5 mm) 34, 78, 79. Whereas [^18^F]JK-PSMA-7 showed similar image resolution relative to urea-based ^18^F-labeled radiotracers, it demonstrated increased PSMA-specific cellular uptake as well as considerably higher imaging acutance as compared to [^18^F]DCFPyL. Furthermore, [^18^F]JK-PSMA-7 demonstrated higher target-to-background ratios as compared to all other radiotracers 34.

**Figure 6:**
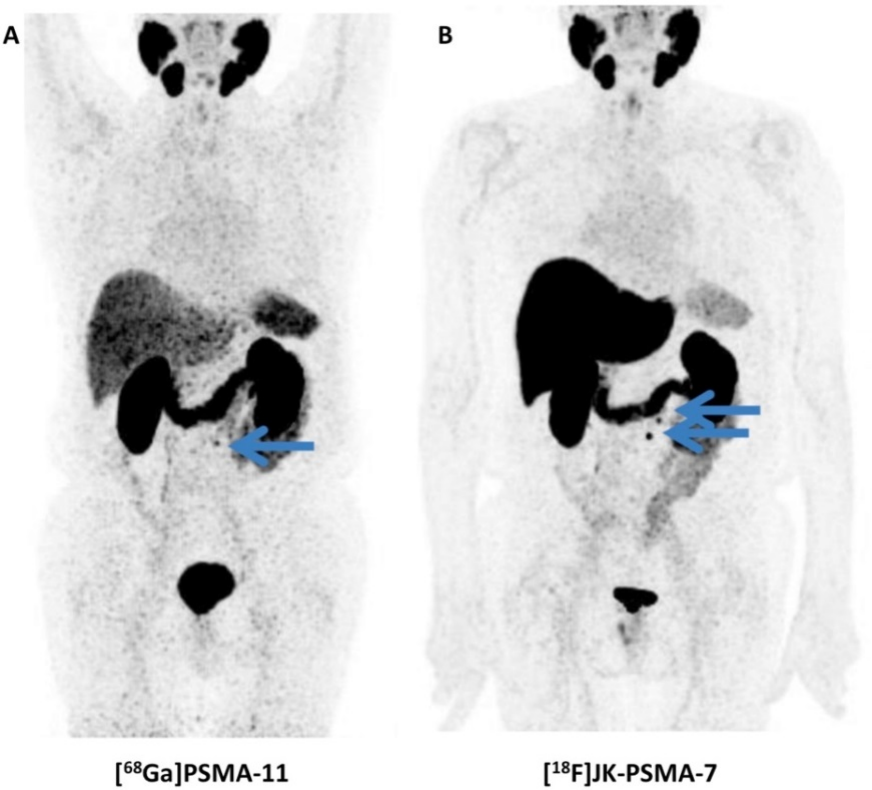
** Head-to-head comparison of [^68^Ga]PSMA-11 vs. [^18^F]JK-PSMA-7.** Whole-body distribution of [^68^Ga]PSMA-11 (A) vs. [^18^F]JK-PSMA-7 (B) in the same patient. [^68^Ga]PSMA-11 revealed only one PSMA-positive retroperitoneal paraaortal lymph node (blue arrow), whereas the [^18^F]JK-PSMA-7 PET/CT showed two PSMA-positive retroperitoneal lymph nodes (blue arrows). Modified from *Dietlein* et al. 109, © by the Society of Nuclear Medicine and Molecular Imaging, Inc.

*Clinical Investigations. In vivo* data has been acquired in a first-in-human study as well as in a biodistribution and dosimetry study (n=10) 80, in a direct comparison with [^68^Ga]PSMA-11 (n=10) and in clinical applications in a larger clinical population (n=124, Figure 6) 81. These studies demonstrate physiologic radiotracer accumulation in a pattern resembling the distribution known from other PSMA-targeted radiotracers with excretion via urinary and biliary pathways. Regarding dosimetry, whole body doses similar to other radiotracers were reported, with a maximum in the kidneys. High uptake in suspicious lesions was found, increasing over time, suggesting benefits of a late start of the PET/CT acquisition 80. A pilot study in 10 patients who were examined with both PSMA-targeted radiotracers demonstrated that [^18^F]JK-PSMA-7 was at least equivalent to [^68^Ga]PSMA-11, as all [^68^Ga]PSMA-11-positive lesions could be seen with [^18^F]JK-PSMA-7 and several additional lesions were detected. In a subsequent analysis of a larger clinical cohort, [^18^F]JK-PSMA-7 was found useful in various diagnostic scenarios (initial staging, biochemical recurrence and therapy monitoring). Notably, in patients with biochemical recurrence after prostatectomy (PSA level, ≥ 0.17 ng/ml) or in patients with biochemical recurrence after radiation therapy (PSA levels, ≥ 2 ng/ml), PSMA-positive lesions were detected in 44/53 patients (83%) 81. Direct comparison with other ^18^F-labeled PSMA-targeted radiotracers appears warranted, in particular as sensitivity for [^18^F]DCFPyL has been described moderately lower in a similar clinical scenario (74.2%) 81.

### [^18^F]AlF-PSMA-11

*Radiochemistry and Preclinical Investigation.* Using a direct labeling procedure of PSMA ligands via aluminum fluoride ^18^F-AlF-complexation, the novel imaging agent [^18^F]AlF-PSMA-11 has been compared to [^68^Ga]PSMA-11 in BALB/c nude mice with PSMA-expressing tumors. While tumor lesions could be appreciated with comparable uptake, [^18^F]AlF-PSMA-11 demonstrated lower renal accumulation, which in turn renders this novel ^18^F-labeled agent as an attractive compound for detection of small lesions close to the urinary tract. However, as a drawback, [^18^F]AlF-PSMA-11 demonstrated a time-dependent increase of radiotracer uptake in the bone, and such defluorination may influence the accuracy of lesion detection in the skeleton 35. The favorable binding affinities to PCa has also been proven by investigating PSMA-high LNCaP vs. PSMA-low PC3 tumors, showing a 24-fold higher uptake for [^18^F]AlF-PSMA-11 in the high LNCaP tumors in C57BL6 mice 82. Notably, a recently established automated synthesis of this compound will also allow for large scale production and gurantaee a high throughput of patients in a busy PET practice 83-85.

*Clinical Investigations.* In a recent dosimetry study, *Piron* and coworkers reported on a high safety profile in six patients with PCa. In addition, [^18^F]AlF-PSMA-11 has a considerable low mean total radiation dose (comparable to [^18^F]DCFPyL) 86. Besides, a recent head-to-head comparison in 15 patients afflicted with PCa using both [^68^Ga]PSMA-HBED-CC and [^18^F]AlF-PSMA-11 showed that in additional 22% of the cases, bone lesions were only discernible with the latter compound 87.

**Figure 7:**
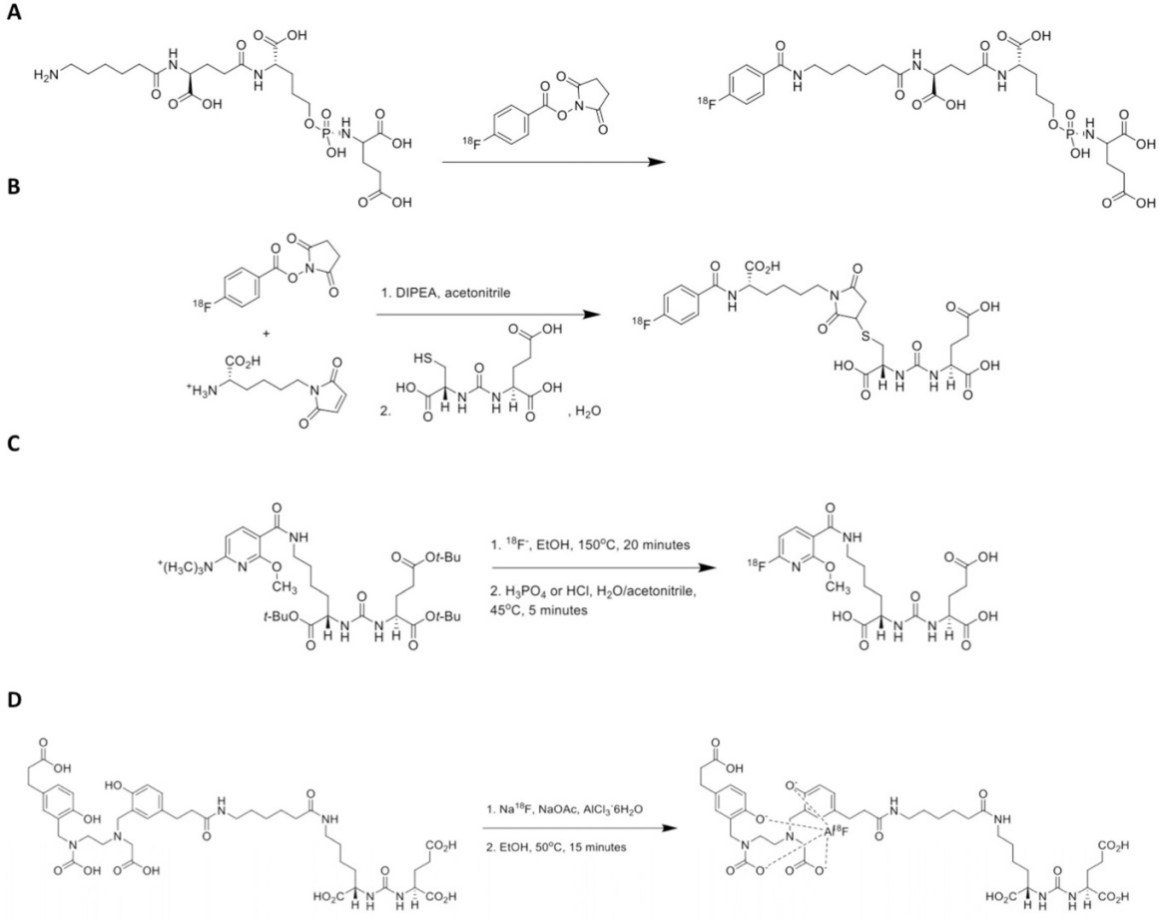
** Scheme showing radiosynthesis procedures for recently introduced PSMA-targeting radiotracers.** (A) [^18^F]CTT1057 (modified from et *Behr* al. 32, © by the Society of Nuclear Medicine and Molecular Imaging, Inc.), (B) [^18^F]FSU-880 (modified from *Harada* et al. 77, © by the Society of Nuclear Medicine and Molecular Imaging, Inc.), (C) [^18^F]JK-PSMA-7 (modified from *Zlatopolskiy* et al. 34, © by the Society of Nuclear Medicine and Molecular Imaging, Inc.) and (D) [^18^F]AlF-PSMA-11 (modified from *Lütje* et al. 35, © by the Society of Nuclear Medicine and Molecular Imaging, Inc.). DIPEA = diisopropylethylamine.

Figure 7 provides schemes showing radiosynthesis procedures for [^18^F]CTT1057, [^18^F]FSU-880, [^18^F]JK-PSMA-7, and [^18^F]AlF-PSMA-11.

### Choosing the appropriate ^18^F-labeled radiotracer

As demonstrated by the herein reviewed large variety of ^18^F-labeled radiotracers, recent years have witnessed a tough competition in PSMA inhibitor imaging, which may be mainly driven by the ease of radiochemistry and the high incidence of PCa compared to other gender-specific tumor entities. Notably, PCa is the most common diagnosed cancer in men, while ovarian cancer ranks 7^th^ of the most commonly diagnosed tumor among women 88. Given the wide array of different ^18^F-labeled compounds for PCa imaging in men, it may be challenging to decide which radiotracer to introduce in one's institution. As outlined above, all of these imaging agents have demonstrated good imaging performance, potentially outperforming current diagnostic tools, e.g. Na^18^F PET, ^68^Ga-PSMA PET/CT, CT, and/or bone scan. However, distinct radiotracer characteristics were noted among these ^18^F-labeled radiotracers. [^18^F]DCFPyL has very low hepatic uptake, which allows for the detection of small liver lesions even when cross-sectional correlates cannot be appreciated 14 or may be of value in later stages of disease 51. [^18^F]PSMA-1007 and [^18^F]AlF-PSMA-11 have very low radiotracer accumulation in the urinary system, which renders these imaging agents an attractive alternative to identify small lesions in the pelvis or for local recurrence 35, 69. [^18^F]JK-PSMA-7 may have higher target-to-background ratios and imaging acutance compared to [^68^Ga]PSMA compounds as suggested by pre-clinical and clinical data 34, 81. [^18^F]CTT1057 has a phosphoramidate core which may allow for irreversible binding to sites of disease and its theranostic twin has already been tested in a preclinical setting 32, 75. Nonetheless, the overall lesion detectability may be substiantially hampered by the different binding affinites and thus, IC50 values may serve as a reliable metric of which agent might generate the necessary radioactivity concentration to identify small volume sites of disease. For instance, [^18^F]DCFPyL has the lowest binding affinity (IC50 = 12.3 nM), followed by [^18^F]PSMA-1007 (IC50 = 4.2 nM), [^18^F]FSU-880 (IC50 = 2.2 nM) and [^18^F]CTT1057 (IC50 = 0.4 nM) 74, 77, 89. Thus, the affinity of the latter agent would be theoretically 30 fold higher compared to [^18^F]DCFPyL (or 10 fold higher compared to [^18^F]PSMA-1007). However, human PSMA-targeted PET imaging is a complex interplay of varying factors including biodistribution, radiotracer accumulation in putative sites of disease, renal excretion or normal variant uptake in benign lesions 90. Thus, it is a matter of debate whether IC50 values derived by cell assays indeed reflect clinical reality 74. In this regard, SUV may serve as a useful tool in clinical practice. *Giesel* et al. reported on slightly higher SUV_max_ values for [^18^F]DCFPyL compared to [^18^F]PSMA-1007 among lesions in different organ compartments without reaching statistical significance (range, [^18^F]PSMA-1007, 10.2 - 17.7 vs. [^18^F]DCFPyL, 11.6 - 18.1) 51. For [^18^F]CTT1057, comparable results have been reported (5.9 - 19.1). Uptake of [^18^F]JK-PSMA-7 has also been found to be in the same range (12.8 ± 7.7) 32, 80. For the latter agent, the tumor-to-background ratios (with the gluteus muscle serving as reference) were significantly elevated, in particular in delayed scans 80 and such ratios would allow for a better comparison of the performance among all available radiotracers. However, considering the varying biodistribution of these agents, a semi-quantitative assessment of the tumor-to-bloodpool ratio, preferably compared to the net influx rate, would be desirable 91.

Table 3 highlights the advantages and limitations of the reviewed ^18^F-labeled compounds.

In light of these distinct characteristics among different ^18^F-labeled PSMA PET agents, an argument could be made that in the era of precision medicine, the use of the right radiotracer depends on what is currently needed by the interpreting nuclear medicine specialist or referring treating physician. Practically speaking, however, it is unlikely that all of these agents will become widely available or that they will all be available at a given site. However, that should not discourage users of other agents to also pursue the necessary multi-center prospective data to submit New Drug Applications for other ^18^F-labeled PSMA-targeted compounds. Regulatory approval should also be sought in other jurisdictions. Ultimately, despite the nuanced differences in these compounds, all of them provide high diagnostic yield and the most-used compound will be that which is most widely available and conveniently accessed for a reasonable cost. Nonetheless, additional comparative studies are needed to firmly establish the role of all available ^18^F-labeled compounds in the field of molecular PCa imaging, preferably in different clinical scenarios, which will allow for determining the true clinical utiltiy and benefit for patients 92. Albeit first results have been recently published 51, such head-to-head comparisons on a larger scale are urgently needed. If not, we have nothing more than an *embarrassment of riches*, or in this case, radiotracers 92.

## Future Perspectives

*Modification of Current Imaging Protocols.* [^18^F]DCFPyL, [^18^F]CTT1057 and [^18^F]FSU-880 have high urinary excretion and, thus, these agents may benefit from modified imaging protocols to improve overall detection rate. These strategies include: intravenous application of furosemide, oral hydration, or voiding prior to imaging 32. All ^18^F-labeled agents have shown better target-to-background ratios in delayed scans and thus, future protocols should not start imaging acquisition prior to 90 min after radiotracer administration 31, 32, 52. Moreover, as suggested by *Schmuck* et al. using [^68^Ga]PSMA I&T, an early dynamic followed by a static delayed image acquisition also leads to an improved tumor-to-nontumor ratio, in particular in the prostate gland 93 and this may be relevant for ^18^F-labeled PSMA PET agents as well.

*Risk Stratification and Application to Theranostics.* For potential use in a theranostic setting, only [^177^Lu]CTT1403, which is based on the same PSMA-binding scaffold as the PET agent [^18^F]CTT1057, has been investigated in a preclinical environment 75. Nonetheless, given the success of RLT based on [^68^Ga]PSMA PET-based imaging, one may expect that ^18^F-targeting PSMA agents will also be used to assess treatment rationale for a therapeutic approach with alpha- or beta-labeled PSMA ligands 12. The field of PSMA-targeted theranostics has been fueled by the prolonged progression-free and overall survival in midgut neuroendocrine tumors treated with ^177^Lu-based somatostatin receptor agonists 8. However, as recently outlined by *Bodei* and coworkers, strategies to predict efficacy should be implemented in the clinic, otherwise nuclear medicine physicians may have to deal with a decline in interest by the referring oncologists, in particular as some patients may suffer from progressive disease under treatment 94. In patients undergoing RLT, up to 20% do not respond with PSA decline and thus, future efforts should turn towards selecting patients most likely to benefit from such an endoradiotherapy 95. Given the tentatively superior diagnostic performance of ^18^F-labeled PSMA-targeted PET agents 18, 19, risk stratification assessments based on these radiotracers may pave the way for a more reliable detection of treatment responders. However, prior to an application in a theranostic setting, ^18^F-labeled PET-based findings should be further validated using histopathological reference, which has already been demonstrated for [^68^Ga]PSMA-11 PET 96, 97.

*Other (pre)clinical competitors in the field of PSMA-directed imaging and treatment.* Apart from the reviewed ^18^F-labeled PSMA-targeted PET agents, other competitors have penetrated the (pre)clinical arena for molecular imaging of PCa. For instance, *Cantiello* et al. reported on the superior performance of [^64^Cu]PSMA-617 (half-life, 12.7 h) compared to [18F]choline in restaging after biochemical recurrence, in particular in subjects with low PSA levels 98. ^89^Zr-labeled monoclonal antibodies specifically designed for immuno PET imaging of PSMA expression have been also investigated in the preclinical and early clinical settings 99-101. Recently, the generator produced radiopharmaceutical [^44^Sc]Sc-PSMA-617 has been investigated and its long half-life of 4.04h renders it as an attractive PET agent for peri-therapeutic dosimetry studies 102. Nonetheless, single photon emission computed tomography (SPECT) is more widely available as PET and PSMA-targeted SPECT compounds, such as the ^111^In-labeled PSMA I&T, are a useful substitute if PET is not available 103. Moreover, this compound has already proven its value in radioguided surgical procedures leading to high intraoperative detection rates of metastatic PCa lesions 104.

## Conclusions

Due to inherent advantages, recent years have witnessed a shift from ^68^Ga-labeled PSMA-targeted compounds towards ^18^F-labeled radiotracers. These include: [^18^F]DCFPyL, [^18^F]PSMA-1007, and four other recently-introduced compounds ([^18^F]JK-PSMA-7, [^18^F]CTT1057, [^18^F]FSU-880 and [^18^F]AlF-PSMA-11). All of these ^18^F-labeled PSMA PET imaging agents have demonstrated good imaging quality, potentially outperforming current imaging modalities. However, further research is warranted, e.g. to elaborate on the benefit of late imaging time-points or improved imaging protocols (e.g. by intravenous application of furosemide).

## Figures and Tables

**Table 1:** Head-to-head comparison of ^68^Ga and ^18^F radiochemistry for prostate cancer molecular imaging.

	Disadvantages	Advantages
^68^Ga	Lower positron yield and longer positron range lead to an increased partial volume effect, which in turn hampers diagnostic accuracy and semiquantitative approaches 37 Limited time-span between injection of the radiopharmaceutical and start of the scan 37 Increased imaging noise compared to ^18^F 37 ^68^Ga-based synthesis allows for the production of sufficient radioactivity for 5 to 6 scans per day (depending on the time point of the generator life span) 36 Large variety of commercially available ^68^Ga generators with varying properties, e.g. different hydrochloric acid concentration or eluate processing procedures 39 Deterioriating performance of ^68^Ge / ^68^Ga generators	Most commonly used radiotracer in clinical practice to date for prostate cancer Easily obtainable via commercially available ^68^Ga generators Extensively tested in large clinical trials, e.g. in a prospective single-arm trial enrolling 635 men 47 Extensively used in a theranostic setting for subsequent ^177^Lu-based therapies 12
^18^F	Installation and maintenance of a costly cyclotron is required 23 Time-consuming and challenging radiosynthesis 23 No larger prospective trials to date (comparable to ^68^Ga-PSMA PET 47) Experience with theranostic approaches based on ^18^F-PSMA PET are limited	Theoretically, it allows for the injection of less radioactivity, which minimizes radiation exposure to both patients and personnel 15 Increases the stability of a radiopharmaceutical against metabolism at sensitive positions and allows for higher flexibility in the study design 15 Allows for delayed imaging protocols, which may increase lesion detection rate (in a manner similar to late imaging protocols using ^68^Ga-labeled PSMA PET radiotracers) 15, 43, 44, 93 Cyclotron-based synthesis of ^18^F allows for the production of larger batches on the basis of a single synthesis 36 Delivery to smaller hospitals operating a PET center remote from a cyclotron facility: -Expands the armamentarium of available PET radiopharmaceuticals in smaller hospitals -Cost-effective alternative (compared to in-house productions), as shown with [^18^F]FDG 41

**Table 2:** ** Comparison of** [^18^F]**DCFPyL and** [^18^F]**PSMA-1007 to ^68^Ga-labeled PSMA-targeting radiotracers.** BR = biochemical recurrence. RP = radical prostatectomy. RT = radiation therapy.

	[^18^F]DCFPyL	[^18^F]PSMA-1007	[^68^Ga]PSMA
Detection rates (benign lesions) on a per-patient based analysis	n/a	Matched pair-analysis with 102 subjects Approximately 5 times more lesions attributed to benign origin compared to ^68^Ga-PSMA-11 (245 vs. 52 lesions) 73	
Overall detection rates (putative sites of disease) on a per-patient based analysis	130 subjects with BR treated with RP (72.3%) or RT (34.6%): 110/130 (84.6%) 53 62 subjects with BR after RP (61%) or RT (39%): 46/62 (74.2%) 19	251 subjects with BR treated with RP (100%): 204/251 (81.3%) 69 100 subjects with BR treated with RP (92%) or RT (45%): 95/100 (95%) 68	635 subjects with BR after RP (41%), RT (27%) or both (32%): 475/635 (75%) 47
PSA levels	130 subjects with BR treated with RP (72.3%) or RT (34.6%) 60%, 78%, 72%, and 92% for patients with PSA levels of ≥0.4 to ≤0.5,≥0.5 to <1.0, ≥1.0 to <2.0, and ≥ 2.0 ng/ml 53 31 patients with biochemical recurrence following radical prostatectomy 59.1% and 88.9% among patients with PSA levels of <1 and >1.0 ng/ml 105	100 subjects with BR treated with RP (92%) or RT (45%) 86%, 89%, 100% and 100% among subjects with PSA levels of ≤0.5, 0.51-1.0, 1.1-2.0, and >2.0 ng/ml 68 251 subjects with BR treated with RP (100%) 61.5%, 74.5%, 90.9%, and 94% among patients with PSA levels of <0.5, 0.51-1.0, 1.1-2.0, and ≥2.0 ng/ml 69	Metaanalysis including 4,970 subjects 33%, 45%, 59%, 75%, and 95% among patients with PSA levels of 0-0.19, 0.2-0.49, 0.5-0.99, 1-1.99 and > 2.0 mg/ml 106 635 subjects with BR after RP (41%), RT (27%) or both (32%) 38%, 57%, 84%, 86%, and 97% among patients with PSA levels of <0.5, 0.5 to <1.0, 1.0 to <2.0, 2.0 to <5.0, and >5.0 ng/ml 47
PET positivity based on Gleason Score (derived by biopsy or prostatectomy)	130 subjects with biochemical recurrence treated with RP (72.3%) or RT (34.6%) ≤6: 13% 7: 50% ≥8: 37% 53, 107	100 subjects with BR treated with RP (92%) or RT (45%) ≤6: 6% 7: 43% ≥8: 28% not specified: 23% 68, 107	Metaanalysis including 1,615 subjects 7: 72% 8: 80% 106
Change in Management after Scan	130 subjects with BR treated with RP (72.3%) or RT (34.6%): 87% 53	n/a	Metaanalysis including 1,163 subjects: 45% 108

**Table 3:** Advantages and limitations of the different ^18^F-labeled compounds for PSMA PET imaging.

	Disadvantages	Advantages
[^18^F]DCFPyL	Reduced binding affinity *in vitro* 89 Clearance via urinary tract for [^18^F]DCFPyL in the first 2h p.i., 11% (vs [^18^F]PSMA-1007, 1.2%) 14, 31	Very low hepatic uptake, which allows for the detection of small liver lesions 14 May be of value in later stages of disease 51 One of the most extensively validated of the ^18^F-labeled radiotracers for PSMA PET imaging Most likely the first of the herein reviewed compounds that will be approved by the U.S. Food and Drug Administration
[^18^F]PSMA-1007	Higher hepatic background, which may be a drawback in later stages of disease (for the detection of liver lesions) 51	Very low radiotracer accumulation in the urinary system, which renders this imaging agent an attractive alternative to identify small lesions in the pelvis or for local recurrence 69
[^18^F]CTT1057	To date, application to different clinical scenarios on a larger scale are still lacking	Phosphoramidate core may allow for irreversible binding to sites of disease 32, 75 Its theranostic counterpart [^177^Lu]CTT1403 has already been tested in a preclinical setting 32, 75 Minimal small bowel activity, i.e. useful to assess sites of disease in the mid-abdomen, e.g. small lymph nodes 32
[^18^F]FSU-880	To date, application to different clinical scenarios on a larger scale are still lacking	Almost exclusive excretion from the kidneys and moderate to low liver uptake 77
[^18^F]JK-PSMA-7		Higher target-to-background ratios and imaging acutance 34, 81 Useful in various diagnostic scenarios (initial staging, biochemical recurrence, therapy monitoring) 81
[^18^F]AlF-PSMA-11	Time-dependent increase of radiotracer uptake in the bone, and such defluorination may influence the accuracy of lesion detection in the skeleton 35	Very low radiotracer accumulation in the urinary system, i.e. useful to identify small lesions in the pelvis recurrence 35
